# Correction: Improved lateral heat spreading performance for polyvinylidene fluoride composite film comprising silver nanowire in light-emitting diode

**DOI:** 10.1039/c8ra90106f

**Published:** 2019-01-04

**Authors:** Zhao Li, Li Zhang, Rong Qi, Fan Xie, Shuhua Qi

**Affiliations:** Department of Applied Chemistry, School of Science, Northwestern Polytechnical University Xi’an 710072 PR China qishuhuanwpu@163.com

## Abstract

Correction for ‘Improved lateral heat spreading performance for polyvinylidene fluoride composite film comprising silver nanowire in light-emitting diode’ by Zhao Li *et al.*, *RSC Adv.*, 2016, **6**, 35844–35891.

The authors wish to apologise to the readers and draw their attention to our closely related paper, published in *Journal of Applied Polymer Science*,^1^ which should have been cited in this *RSC Advances* paper.

In the *RSC Advances* paper, we report a lateral heat spreader film fabricated by silver nanowire (AgNW) and polyvinylidene fluoride (PVDF) film *via* a bar-coating approach. In order to distinguish the composite film in this paper from that in our previous work,^1^ we are renaming the composite film as “AgNW@AgNW/PVDF”, which indicated that AgNW was coated on the AgNW/PVDF film. In this work, the results come from the AgNW@AgNW/PVDF composite film, while in our previous work,^1^ they come from the AgNW/PVDF film, which are fundamentally different. In ref. 1, we used AgNW as an additive to a PVDF/DMF precursor, and then synthesized a AgNW/PVDF composite film for heating transfer. The work focused on the improvement of the thermal conductivity of the film; however, its application was never mentioned.

In this paper, AgNW was added into PVDF as an additive, but it was also coated on the surface of the AgNW/PVDF film by a bar-coating approach. Thus, this work can promote the thermal conductivity from inside and outside due to the high thermal conductivity of AgNW. Additionally, we set up a temperature monitoring system using the infrared imager, which can capture the temperature image while the composite film is being heated. We compared experimental with theoretical analysis by simulation (ANSYS Icepak) of the physical process, and they matched very well. Finally, the AgNW@AgNW/PVDF composite film was used as a lateral heat spreader in a LED device that we devised. The good result obtained from detecting the temperature of the LED by thermometer showed its excellent heat dissipation performance.

In addition to the lacking citation to the *Journal of Applied Polymer Science* paper, the authors also regret that there is unattributed overlap in text and Fig. 1, 3a, c, 4, 6b and c between this *RSC Advances* paper and ref. 1. The figures were reproduced from ref. 1 for the readers’ information.

The Royal Society of Chemistry apologises for these errors and any consequent inconvenience to authors and readers.

## Supplementary Material
